# A microbiological evaluation of level of disinfection for flexible cystoscopes protected by disposable endosheaths

**DOI:** 10.1186/1471-2490-13-46

**Published:** 2013-10-07

**Authors:** Peter Hjorth Jørgensen, Torsten Slotsbjerg, Henrik Westh, Vicki Buitenhuis, Gregers Gautier Hermann

**Affiliations:** 1 Department of Urology, Frederiksberg University Hospital, Frederiksberg, Denmark; 2 Department of Clinical Microbiology, Hvidovre University Hospital, Hvidovre, Denmark; 3 Faculty of Health and Medical Sciences, University of Copenhagen, Copenhagen, Denmark

**Keywords:** Flexible cystoscopy, Endosheaths, Microbiological assessment, Disinfection, Bladder cancer

## Abstract

**Background:**

Flexible cystoscopy is used in urological outpatient departments for diagnostic cystoscopy of bladder cancer and requires a high-level disinfection between each patient. The purpose of this study was to make a microbiological post disinfection efficacy assessment of flexible cystoscopes (FC) using disposable sterile endosheaths.

**Methods:**

One hundred endosheaths underwent a leak-test for barrier integrity after cystoscopy. Microbiological samples from these cystoscopies were obtained; after removal of the endosheath, and after cleaning the scope with a detergent cloth, rinsing with tap water followed by 70% ethanol disinfection and subsequent drying. The number of colony forming units (cfu) from the samples was counted after 72 hours and then divided in three categories, Clean FC (<5 cfu/sample), Critical FC (5–50 cfu/sample) and High-risk FC (>50 cfu/sample). The result was compared with data of 10 years continuous control sampling recorded in the Copenhagen Clean-Endoscope Quality Control Database (CCQCD) and analyzed with a Chi-square test for homogeneity.

**Results:**

All 100 endosheaths passed the leak-test. All samples showed a Clean FC and low means of cfu. A query to the CCQCD, showed that 99.8% (1264/1267) of all FC with a built-in work-channel reprocessed in a WD were clean before use.

**Conclusion:**

The reprocessing of FC using endosheaths, as preformed in this study, provides a patient-ready procedure. The results display a reprocessing procedure with low risk of pathogen transmission, high patient safety and a valid alternative to the recommended high-level disinfection procedure of FC. However, the general impression was that sheaths slightly reduced vision and resulted in some patient discomfort.

## Background

Flexible cystoscopy is a common procedure in Danish urological outpatient departments (OPD). Based on the Danish Bladder Cancer Registry, up to 20 000 cystoscopies are performed each year for either diagnostic purposes or as part of post-surgery follow-up of bladder tumors (BT) [1]. FC can be classified as semi-critical or critical devices, and therefore a high-level disinfection between each patient has been suggested [2]. No general guidelines for reprocessing flexible endoscopes (FE) include FCs. The recommended standard disinfection of FCs consist of manual cleaning followed by rinsing and drying in a washer disinfector (WD) and finally flushing of the FC work-channel with alcohol. This practice is time-consuming as well as relatively expensive and requires specific equipment, facilities and trained staff. It is therefore of importance to develop and evaluate new approaches concerning this everyday urological procedure.

Studies on FCs with or without the use of disposable polymer endosheaths have been made. All have focused on optical quality, procedure time, patient comfort and financial aspects [3-6]. The present study is to our knowledge, the first to evaluate bacterial contamination of cystoscopes protected by endosheaths and the efficacy of reprocessing. Data on bacterial contamination in relation to level of disinfection are necessary where implementing new FC procedures in clinical use.

## Methods

We included 100 patients who underwent flexible cystoscopy in the OPD, Frederiksberg Hospital, Denmark in the follow up of BT from August to December 2010. Only one cystoscope was used (Model CST_4000, Vision^®^ Sciences, Inc.; Orangeburg, NY, USA) covered by the sterile single use Slide-On Endosheath^®^ System with the built-in work-channel in the endosheath (EndoSheath; Vision^®^ Sciences Inc.; Orangeburg, NY, USA).

After cystoscopy, cultures were obtained immediately after removal of the sheath, using sterile saline pledgets (Sterile Wipes PDI^®^, Inc. Orangeburg, NY, US), from the maneuver part and the insertion part of the cystoscope, as described by Alvarado et al [7]. Then, the cystoscope was wiped with a detergent cloth (Wet Wipe^®^ A/S; Vallensbæk, Denmark), rinsed with running tap water, manually dried off with gauze, wiped with 70% ethanol soaked gauze and finally hung vertically for the ethanol to evaporate. Post disinfection cultures were also obtained, after 10 minutes drying.

The sample pledgets were placed in 1 ml sterile saline and shaken for 30 seconds. Ten drops of 0.02 ml aliquots from the sample were spotted on two 5% blood agar plates (Statens Serum Institut, Denmark) and incubated at 35°C in CO^2^. The total number of colony forming units (cfu) was counted after 72 hours. Bacteria were speciated using standard methods.

The endoscope was visually inspected after use for contamination with body fluids. All 100 sheaths underwent a test for barrier integrity post cystoscopy using a Leak Testing/Pressure Decay Equipment (Vision^®^ Sciences Inc., Orangeburg, NY, USA).

After sampling the cystoscope was reprocessed according to standard operating procedures in a WD.

As in the Copenhagen Clean-Endoscope Quality Control Database (CCQCD) we defined three grading levels of reprocessing FC. Clean FE (<5 cfu/sample), Critical FE (5–50 cfu/sample) and High-risk FE (>50 cfu/sample).

The disinfection quality level of FC after traditional reprocessing and FC with sterile endosheaths with a built-in work-channel were compared.

The results of samples obtained before and after reprocessing the FC were analyzed for homogeneity with a Chi-square test, based on means of cfu.

The study was performed in accord to the Helsinki Declaration and regulations of the local ethical committee and was regarded as a quality performance project and all patients participating in the procedures, were adults and had given their oral and written accept.

## Results

All samples obtained after removal of the sheath and after cleaning and alcohol disinfection, both from the maneuver part and insertion part of the FC showed a Clean FC with a mean of 0.2 cfus (Table 1). Table 1 shows that, samples from the FC before and after reprocessing demonstrated no significant reduction of cfus by reprocessing. The bacteria identified were coagulase negative staphylococci, Bacillus species and Corynebacterium species.

**Table 1:** Microbiological samples from 100 cystoscopies preformed with a Vision Sciences FC (Model CST 4000; Vision^®^ Sciences, Inc.), after removal of the endosheath and after cleaning the FC with a detergent cloth, rinsing with tap water followed by 70% ethanol disinfection and subsequent drying

		**Maneuver part**		**Insertion tube**	
		**ARS**	**AD**	**ARS**	**AD**
No. of samples		100	100	100	100
No. of cfu/sample					
**Clean FC**	0	82	87	88	87
	1	12	11	9	9
	2	4	1	3	3
	3	2	1	0	0
	4	0	0	0	1
**High-risk/critical FC**	>5	0	0	0	0
Mean cfu/sample (NS)		0.26	0.16	0.15	0.19

*ARS* After removal of the sheath, *AD* After disinfection, *NS* Non-significant.

All 100 endosheaths passed the leak-test after the cystoscopy and no endoscopes were visibly contaminated with body fluids.

A query to the CCQCD, showed that 99.8% (1264/1267) of all FC with a built-in work-channel reprocessed in a WD were clean before use.

## Discussion

After using FCs with disposable sterile endosheaths, the ensuing cleaning and disinfection of the FCs serves two purposes. The first is to clean the endoscope in case of failure of antiseptic technique by the user. The second is to mechanically remove material that may have been deposited by the insertion tube or onto the scope while applying or removing the sheath.

The use of endosheaths in this study ensured that no body fluids came in contact with the scope, making it possible to do the manual cleaning with a pre-packed single use cloth, moistened with a non-enzymatic detergent in the examination room.

Prevention of contamination with bacteria from the patient and environment is reliably prevented with 70% ethanol. Furthermore 70% ethanol protects against most viruses (except, for instance HPV) and evaporates water leaving the FC dry. Being dry before reuse, it is now possible to do a “leak test” by inspecting the scope-surface for moisture after removal of the next sheath. If the surface of the FC was wet after removal of the endosheath, it could indicate a leak and that direct contact between mucosa and cystoscope had taken place.

The few bacteria (1 to 4 cfus) we identified originated from skin commensals, and were found in a few samples taken both before and after reprocessing the FC. As no leak was detected, the contamination is presumed to come from the handling of the FC or from sampling procedures.

A study by Alvarado et al. [7] showed clean flexible nasopharyngoscopes (FN) after they were desheathed and cleaned with enzymatic detergent followed by 70% ethanol disinfection and evaporation. They developed the method used in our study, and even though a FC is longer than a FN, we only found a few cases of contamination with the same environmental microorganisms. This indicates that our method of reprocessing the FC, without the use of enzymatic detergent, was reliable. Should a leak occur, it will be revealed by the presence moisture on the FC after removal of the sheath. This contaminated FC, which had contact with body, must be processed by manual cleaning with an enzymatic detergent followed by a high level disinfection in a washer desinfector [2,8]. This setting requires a separate cleaning room with a basin with a detergent solution to soak and manual clean the FC.

The quality of the disposable sheaths used was high. In a prior study with 875 endoscopic procedures, no leak has been reported [7]. We used sheaths approved by the USA FDA, that are resistant to microorganisms as small as 27 nm and are made out of material with high integrity, unlikely to tear or leak, in contrary to other commonly clinically used barriers [9]. An evaluation of sheaths as a viral barrier has shown a low risk of viruses from contaminated scopes penetrating micro holes and tears in the sheath [10].

The risk of a urinary tract infection (UTI) after FC is estimated to be < 2% to 7.5% [3]. Diagnosing UTI in relation with FC can be difficult due to hematuria, urge, dysuria and leucocyturia, all symptoms that can occur with or without the presence of bacteriuria. In the reported cases of UTIs found in relations with FCs, it has primarily been endogenous bacteria, presumably the patient’s own microorganisms [11].

Exogenous FC related infections appear to be very rare in cystoscopy. Only two major outbreaks have been reported, with UTIs caused by Pseudomonas aeruginosa as a consequence of insufficient reprocessing of the FC. Other studies have showed a correlation between outbreaks and previously damaged or poorly decontaminated instruments [12].

Current USA Multisociety Guideline on Reprocessing Flexible GI Endoscopes, 2011, does not address reprocessing of FCs and FBs [8].In Denmark only automatic decontamination in a WD is recommended when reprocessing FE. Compiled data from the CCQCD has shown a constant low risk of contaminated FC, after manual cleaning including brushing of the work-channel followed by a high level disinfection in an automatic WD. This suggests a differentiation of the guidelines of reprocessing FEs. In comparison with other types of endoscopes, the decontamination of FCs is less complicated because they are single channel instruments. When using FCs with endosheaths, the work-channel is placed in the sheath and the endoscope will generally only become contaminated from handling.

This study did not look into optical quality or patient comfort. In a retrospective overview of the post procedure descriptions and when asking the urologist who performed the cystoscopies, poor optical quality and handling problems were mentioned. Our general impression was that the sheaths slightly reduced vision and resulted in some discomfort for the patients as compared to FC without sheaths. A recent published trial by Krebs et al. assigned 97 patients in a control group with unsheathed FC and a group undergoing sheathed FC (EndoSheath System). The Sheathed procedure saved between four to 31 minutes of reprocessing time, while avoiding exposure to irritants found in conventional soaking methods. The control group scored better than sheath group regarding to insertion of FC, general handling and rinse-water setup (P ≤ 0, 01). No significant difference was found between the two groups comparing procedure time, optical quality and patient comfort both before and after the procedure [4]. Three non-comparative studies reported a minor disadvantage in sheathed FC concerning handling and set up [4-6]. The process of applying and removing the sheath on the FC took approximately one to two minutes.

The current marked prize in Denmark is about $49 per sheath (2012), but most OPDs would be required to change their FCs to FCs compatible with an endosheath system. Nevertheless, when looking at the economical aspect of using sheaths for FCs, the cost of high level disinfecting and staff performing manual cleaning must be considered in the equation [4].

## Conclusion

The reprocessing of FC using endosheaths, as preformed in this study, provides a patient-ready procedure. The results display a reprocessing procedure with low risk of pathogen transmission, high patient safety and a valid alternative to the recommended high-level disinfection procedure of FC. However, the general impression was that sheaths slightly reduced vision and resulted in some patient discomfort.

## Competing interests

The Foundation Juchum and the Boemske Foundations have provided financial support for this study.

## Authors’ contributions

PHJ is first author on the article and has been instrumental in the connection of the urological and microbiological parts of the study as well as being involved in the clinical execution of the flexible cystoscopies. TS is responsible for the conception and design of the study, acquisition of data, analysis of data as well as drafting the primary manuscript. TW has been an important key person behind intellectual content of the microbiological part and critically reviewer of the final draft of the manuscript. VB has been involved is the practical setup and execution of the study. She has been clinical involved in the flexible cystoscopies and collection of data. GGH is responsible for the intellectual content of the urological part of the study as well being the connecting and coordinating person throughout the process. He has given final approval of this version to be published. All authors read and approved the final manuscript.

## Pre-publication history

The pre-publication history for this paper can be accessed here:

http://www.biomedcentral.com/1471-2490/13/46/prepub
